# Supplementary material for: Stringent Response Factor DksA Contributes to Fatty Acid Degradation Function to Influence Cell Membrane Stability and Polymyxin B Resistance of Yersinia enterocolitica
Source: Int J Mol Sci. 2023 Jul 26;24(15):11951. doi: 10.3390/ijms241511951 (PMC10418728; doi:10.3390/ijms241511951)
Supplement: Supplementary file 1 [file ijms-24-11951-s001.zip › ijms-2497357-supplementary.pdf]

Stringent response factor DksA contributes to fatty acid degradation function to influence cell membrane stability and polymyxin B resistance of *Yersinia enterocolitica*

Can Huang[1], Wenqian Li[1], Jingyu Chen[1]*

1 Beijing Laboratory for Food Quality and Safety, College of Food Science & Nutritional Engineering, China Agricultural University, Beijing 100083, China

* Corresponding author: Jingyu Chen

Address: 17 Qinghua East Rd., Beijing 100083, China

E-mail: chenjy@cau.edu.cn

Supplementary Table S1 Primers used in this study

Primers name	DNA Sequences (5' to 3')	Application
16s rRNA-F	GCACGTAATGGTGGGAACTC	RT-qPCR
16S rRNA-R	CTCCAATCCGGACTACGACA	RT-qPCR
YZ-*fadA*-F	AGTACCCGCCAGCCA	RT-qPCR
YZ-*fadA*-R	GAGCAGCAAACGCCT	RT-qPCR
YZ-*fadJ*-F	TGCAACAAGCACAGGCCTTG	RT-qPCR
YZ-*fadJ*-R	GATATCAGCACCAGCGATAAA	RT-qPCR
YZ-*fadE*-F	ACTGCGCCAATCCCT	RT-qPCR
YZ-*fadE*-R	GGTTCCCGCATCAATCGC	RT-qPCR
YZ-*fadD*-F	AGATGTGGTGGCGTTACAT	RT-qPCR
YZ-*fadD*-R	TTACTGTCTCGCCGGAAACC	RT-qPCR
YZ-*fadR*-F	GACAGCGTGCCACAATTGAT	RT-qPCR
YZ-*fadR*-R	GCGTTATCGTCAACCGTCTG	RT-qPCR
YZ-*fadI*-F	CCCTTTGCCAAGCAGGCTAC	RT-qPCR
YZ-*fadI*-R	TCAATCAATTCAGGAGCAAC	RT-qPCR
YZ-*fabA*-F	TGGGCCTTGATGCCATGT	RT-qPCR
YZ-*fabA*-R	CTGGCAGGACTTGC	RT-qPCR
YZ-*fabB*-F	GAATTTAAAGACGCAGGCATG	RT-qPCR
YZ-*fabB*-R	AAGCGCAGCACTTTACGGTC	RT-qPCR
YZ-fabD-F	GTGGCAGTTAGTGCAACAAG	RT-qPCR
YZ-*fabD*-R	CTGCCAAACGCGCCAGATAG	RT-qPCR
YZ-*fabF*-F	GCCAGTAGGCGATCTCGG	RT-qPCR
YZ-*fabF*-R	GACCAGTCGCTGACTTAGT	RT-qPCR
YZ-*fabY*-F	GATGCTTATGATGCAATGGC	RT-qPCR
YZ-*fabY*-R	TCTCACTGATGGGTCTACCG	RT-qPCR
YZ-*fabR*-F	TTTGCGGGAGGTCTCCCG	RT-qPCR
YZ-*fabR*-R	CGCTTTCGTCGACCATTGTC	RT-qPCR
YZ-*fabG*-F	TATTGGTACTGCGACCAGTG	RT-qPCR
YZ-*fabG*-R	CTCGATTGACGCGGGATCCA	RT-qPCR

Supplementary Table S2 Growth gap between YEND sand WT when exposed to polymyxin B

Time (h)	0 MIC	1/2 MIC	1/4 MIC	1/8 MIC
10	6.55%	6.80%	5.93%	5.36%
20	11.36%	19.52%	12.03%	11.27%
30	15.88%	20.35%	17.35%	16.15%
40	10.24%	16.91%	13.09%	11.49%
50	7.82%	10.66%	9.49%	8.93%

Growth Gap = (biomass of WT-biomass of YEND)/ biomass of WT

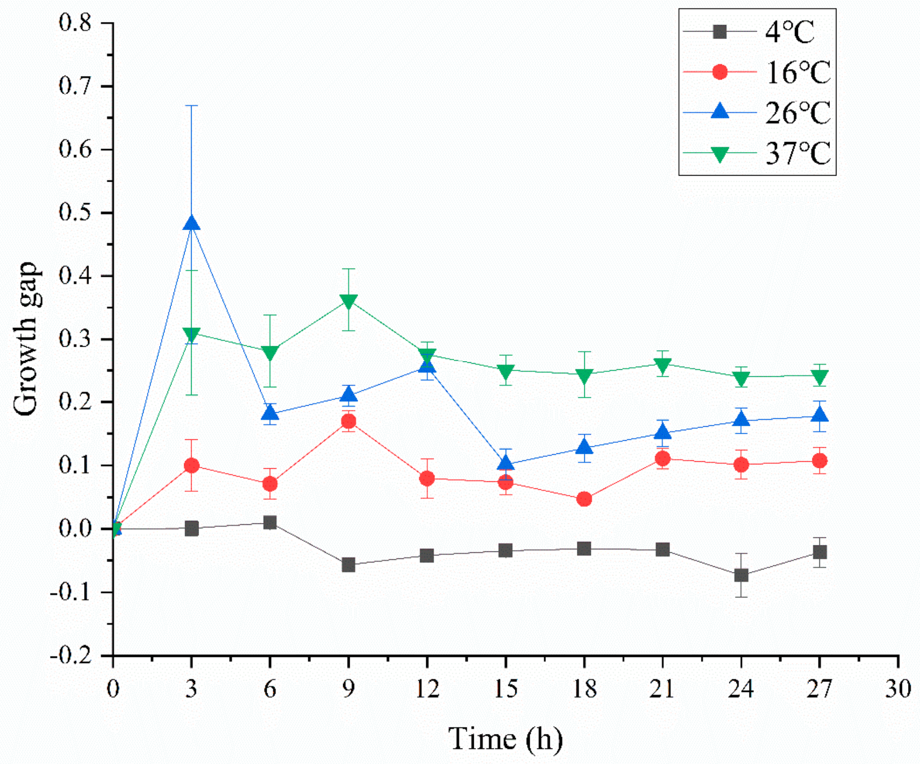

Supplementary Figure S1 Growth gap between YEND strain and WT strain at different temperatures. Growth Gap = (biomass of WT-biomass of YEND)/ biomass of WT, Data are mean of growth gap for three independent experiments and standard errors of the means.
